# Type 2 diabetes exacerbates changes in blood pressure-independent arterial stiffness: cross-sectional and longitudinal evidence from the SUMMIT study

**DOI:** 10.1152/japplphysiol.00283.2023

**Published:** 2023-11-16

**Authors:** Kunihiko Aizawa, Phillip E. Gates, David M. Mawson, Francesco Casanova, Kim M. Gooding, Suzy V. Hope, Isabel Goncalves, Jan Nilsson, Faisel Khan, Helen M. Colhoun, Andrea Natali, Carlo Palombo, Angela C. Shore

**Affiliations:** ^1^Diabetes and Vascular Medicine Research Centre, NIHR Exeter Clinical Research Facility, University of Exeter Medical School, Exeter, United Kingdom; ^2^Department of Clinical Sciences Malmö, Lund University, Malmö, Sweden; ^3^Department of Cardiology, Skåne University Hospital, Malmö, Sweden; ^4^Division of Systems Medicine, University of Dundee, Dundee, United Kingdom; ^5^Centre for Genomic and Experimental Medicine, University of Edinburgh, Edinburgh, United Kingdom; ^6^Department of Clinical and Experimental Medicine, University of Pisa, Pisa, Italy; ^7^Department of Surgical, Medical, Molecular and Critical Area Pathology, University of Pisa, Pisa, Italy

**Keywords:** aging, aorta, blood pressure, ultrasound

## Abstract

Greater central artery stiffness is observed in people with type 2 diabetes (T2DM). Elevated blood pressure (BP) and altered arterial wall structure/composition in T2DM are generally considered as main drivers for this alteration. However, because conventional arterial stiffness measures are BP-dependent and as such an influence of BP remains in a measure, it is unclear if greater central artery stiffness is a function of greater BP, or due to changes in the structure and composition of the arterial wall. We aimed to measure BP-independent arterial stiffness (β_0_) cross-sectionally and longitudinally in T2DM. We studied 753 adults with T2DM (DM+) and 436 adults without (DM−) at baseline (*Phase 1*), and 310 DM+ and 210 DM− adults at 3-yr follow-up (*Phase 2*). We measured carotid-femoral pulse wave velocity and used it to calculate β_0_. In *Phase 1*, β_0_ was significantly greater in DM+ than DM− after adjusting for age and sex [27.5 (26.6–28.3) vs. 23.6 (22.4–24.8) au, *P* < 0.001]. Partial correlation analyses after controlling for age and sex showed that β_0_ was significantly associated with hemoglobin A1c (*r* = 0.15 *P* < 0.001) and heart rate [(HR): *r* = 0.23 *P* < 0.001)] in DM+. In *Phase 2*, percentage-change in β_0_ was significantly greater in DM+ than DM− [19.5 (14.9–24.0) vs. 5.0 (−0.6 to 10.6) %, *P* < 0.001] after adjusting for age, sex, and baseline β_0_. β_0_ was greater in DM+ than DM− and increased much more in DM+ than in DM− over 3 yr. This suggests that T2DM exacerbates BP-independent arterial stiffness and may have a complemental utility to existing arterial stiffness indices.

**NEW & NOTEWORTHY** We demonstrate in this study a greater BP-independent arterial stiffness β_0_ in people with type 2 diabetes (T2DM) compared to those without, and also a greater change in β_0_ over 3 yr in people with T2DM than those without. These findings suggest that the intrinsic properties of the arterial wall may change in a different and more detrimental way in people with T2DM and likely represents accumulation of cardiovascular risk.

## Graphical abstract

**Figure:**
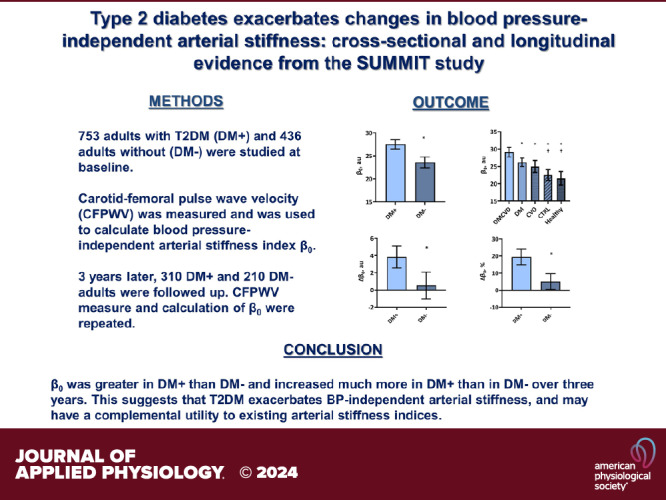

## INTRODUCTION

The stiffness of large elastic arteries is an important property of the cardiovascular system, and a substantial body of research has elaborated its widespread implications for health (1). People with type 2 diabetes (T2DM), for instance, have a greater carotid-femoral pulse wave velocity (CFPWV) than those without (2, 3). Furthermore, CFPWV possesses a prognostic utility for predicting future cardiovascular events in T2DM independently of conventional cardiovascular risk factors (4, 5). The greater CFPWV in T2DM is attributed to the elevated blood pressure (BP) as well as alterations in structure and composition of arteries. However, because CFPWV is inherently BP-dependent (6), it is difficult to interpret CFPWV data cross-sectionally where BP is different between cohorts, as well as experimentally and longitudinally where an intervention reduces BP or changes in BP are observed at follow-up.

To make a situation complicated in T2DM, concomitant hypertension is common (7) and when a therapy alters CFPWV and BP, it is difficult to differentiate if the change is due to a change in BP alone or genuine changes occurring in the intrinsic properties of the arterial wall (8).

There is some circumstantial evidence that shows the alteration in intrinsic properties of the arterial wall in T2DM. For example, hyperglycemic exposure is thought to increase the formation of advanced glycation end-products (AGEs) in the arterial wall, cross-linking collagen and other molecules and increasing the material stiffness of the wall (9). Arterial wall geometry is also altered in T2DM. Common carotid artery intima-media thickness is greater in people with T2DM (10) and because the lumen diameter is preserved (11), wall-to-lumen ratio (WLR) must be greater. This remodeling and structural alteration to the arterial wall could, in turn, increases intrinsic arterial wall stiffness. However, the direct evidence in T2DM is scarce; it is unclear whether BP-independent arterial stiffness is greater in people with T2DM when compared with those without, and it is also unclear whether its progression and deterioration over time are greater in people with T2DM.

Kawasaki et al. proposed the pressure-independent arterial stiffness index β (12) based on the exponential relationship observed experimentally between arterial pressure and diameter within the physiological range (13). Since then, β has been used to quantify intrinsic arterial wall stiffness, for example, to delineate the effects of aging and the responses to lifestyle changes in humans (14, 15). However, an important caveat of β is that it uses diastolic BP in the calculation instead of reference BP, and thus retains a degree of pressure-dependency, albeit less so than conventional arterial stiffness indices. Leaders in the field have explicated the problem and recently provided a solution to correct the pressure-dependency of β, deriving a BP-independent arterial stiffness index, called β_0_ (16).

In this study, we aimed to use β_0_ as a BP-independent index of arterial stiffness in people with and without T2DM using a cross-sectional (*Phase 1*) and longitudinal (*Phase 2*) study design. Our hypotheses were that β_0_ would be greater in people with T2DM than without in *Phase 1*, and that change in β_0_ would be greater in people with T2DM in *Phase 2.* In *Phase 1*, we explored associations of β_0_ with markers of arterial wall material stiffness [hemoglobin A1c (HbA1c) as a surrogate for AGEs] and geometrical alteration (carotid artery WLR) in addition to BP and heart rate (HR) that are known to influence arterial stiffness. Associations of β_0_ with inflammatory markers were also explored. CFPWV was used as a conventional, BP-dependent index of arterial stiffness to present alongside β_0_ and identify any differences between BP-dependent and BP-independent arterial stiffness.

## MATERIALS AND METHODS

### Participants

This is an ancillary analysis of the Surrogate markers for Micro- and Macrovascular hard endpoints for Innovative diabetes Tools-Vascular Imaging Prediction (SUMMIT-VIP) study. The details of the main study have been reported elsewhere (10, 17). Older adults who had been recruited for the SUMMIT-VIP study from Dundee, Exeter (both United Kingdom), Malmö (Sweden), and Pisa (Italy) sites, and whose CFPWV data were available were included in this analysis. For *Phase 1*, we included data collected at baseline from 753 older adults with T2DM (DM+) and 436 without T2DM (DM−). For *Phase 2*, we included data collected both at baseline and following a 3-yr follow-up period from 310 older adults in the DM+ group and 210 in the DM− group. T2DM was defined based on contemporary or historical evidence of hyperglycemia (according to the World Health Organization 1998 criteria: fasting plasma glucose >7.0 mmol/L or 2-h plasma glucose >11.1 mmol/L, or both) or by current medication with insulin, sulfonylureas, metformin, or other antidiabetic drugs. Participants diagnosed with T2DM <35 yr of age or treated with insulin within 12 mo of diagnosis were not included in the study. All study procedures were approved by local ethical review boards and written informed consent was obtained from all participants.

### Carotid-Femoral Pulse Wave Velocity and Calculation of β_0_

The details of our CFPWV acquisition method have been described previously (10, 18). Briefly, the ECG-gated carotid and femoral pulses were recorded sequentially using a commercially available device (SphygmoCor ver 8.2; AtCor Medical, Sydney, New South Wales, Australia). The time difference between the R-wave of the ECG and foot of the carotid and femoral pulse waves was calculated using the intersecting tangent algorithm. Those carotid and femoral pulse waves that meet the quality control criteria were accepted. The arterial path length was obtained by the 4-point method (the sum of the distances between the suprasternal notch and the umbilicus and between the umbilicus and the femoral site, with the distance between the carotid artery and suprasternal notch being subtracted). The recording was repeated three times and the average of those recordings was used for statistical analysis.

β_0_ was calculated using CFPWV according to the method described by Spronck et al. (16):

$$\beta_{0}=\frac{CFPWV^{2}\cdot 2\rho }{Pd}-\ln\;\left(\frac{Pd}{Pref}\right)$$

where CFPWV is the carotid-femoral pulse wave velocity, ρ is the blood mass density, *Pd* is the brachial diastolic BP, ln is the natural logarithm, and Pref is the reference pressure set at 100 mmHg. Blood pressure, CFPWV, and ρ were entered into the equation in units of Pa, m/s, and kg/m^3^, respectively, to ensure dimensional correctness.

### Cardiovascular Risk Factors and Common Carotid Artery Wall-to-Lumen Ratio

Height, body mass, and body mass index were obtained using a standard protocol. Total cholesterol, HDL cholesterol, and HbA1c were measured from participants’ fasting blood samples in each study center. Brachial systolic/diastolic BP and HR were measured using an automated oscillometric device (M5-I; Omron, Kyoto, Japan) three times, and the average of the last two measurements was used for analysis. Mean arterial pressure (MAP) was calculated by adding a third of pulse pressure to diastolic BP. Estimated glomerular filtration rate (eGFR) was calculated using the Chronic Kidney Disease Epidemiology Collaboration creatinine equation (19).

Common carotid artery intima-media thickness and lumen diameter were obtained using an ultrasound machine with a high-resolution linear array transducer, as described previously (20). WLR was calculated as the ratio of common carotid artery intima-media thickness to lumen diameter. In this study, the right common carotid artery WLR was used for statistical analysis as CFPWV was obtained from this side.

### Plasma Markers of Inflammation

As previously described (21), interleukin 6 (IL-6), Chemokine ligand 3 (CCL3), and Pentraxin 3 were obtained using the Proseek Multiplex CVD^96×96^ reagents kit (Olink Bioscience, Uppsala, Sweden) at the Clinical Biomarkers Facility, Science for Life Laboratory, Uppsala, Sweden. All samples were analyzed in the same run. Data analysis was performed by a preprocessing normalization procedure using Olink Wizard for GenEx (Multid Analyses, Gothenburg, Sweden). Values are presented as arbitrary units. Data regarding intra- and inter-assay variations as well as general calibrator curves to calculate the approximate concentrations are available on the OLINK home page. High-sensitivity C-reactive protein (hsCRP) was measured by an immune-turbidimetric method at the Department of Clinical Chemistry at Skåne University Hospital, Malmö, Sweden (22).

### Statistical Analysis

Data are presented as means ± SD, median (interquartile range), means [95% confidence intervals (CI)] or number (%). Skewed data were appropriately transformed for statistical analysis. A Chi-squared test was used to examine the differences in categorical variables. Independent samples *t* tests and analysis of covariance were used as appropriate to examine the differences in variables at baseline and following the 3-yr follow-up period between cohorts. Partial correlation analyses were performed to examine the associations of β_0_ and CFPWV with HbA1c, WLR, MAP and HR, after including age and sex as control variables. Statistical analysis was conducted using IBM SPSS Statistics 28 (IBM, Armonk, NY) and statistical significance was set at *P* < 0.05 (two-sided).

## RESULTS

### Phase 1

Table 1 shows the selected characteristics of the study participants between DM+ and DM−. There were fewer females in DM+ than DM− (*P* = 0.025). The prevalence of pharmacological hypertensive treatment and statin treatment was greater in DM+ than DM− (both *P* < 0.001). Body mass was greater in DM+ than in DM−, as was body mass index (both *P* < 0.001). Total and HDL cholesterol were lower in DM+ than in DM− (both *P* < 0.001). Systolic BP and resting HR were higher in DM+ than in DM− (both *P* < 0.001). Both common carotid artery intima-media thickness (*P* = 0.004) and lumen diameter (*P* < 0.001) were greater in DM+ than in DM−, but WLR was similar between the groups.

**Table 1.** Selected characteristics of the study participants with and without type 2 diabetes

	DM+ (*n* = 753)	DM− (*n* = 436)	*P*
Age, yr	67.5 ± 8.3	67.0 ± 9.2	0.356
Female, *n* (%)	227 (30.2)	159 (36.5)	0.025
History of CVD, *n* (%)	327 (43.4)	200 (45.9)	0.413
HTRx, *n* (%)	578 (76.8)	250 (57.3)	<0.001
Statin, *n* (%)	542 (72.0)	222 (50.9)	<0.001
Current smoking, *n* (%)	83 (11.0)	35 (8.0)	0.092
Duration of T2DM, yr	8 (4–14)		
*Diabetes medications*			
Thiazolidinediones, *n* (%)	47 (6.2)		
Metformin, *n* (%)	506 (67.2)		
Sulfonylureas, *n* (%)	220 (29.2)		
DPP-4 inhibitors, *n* (%)	77 (10.2)		
GLP-1 analogs, *n* (%)	39 (5.2)		
Insulin, *n* (%)	159 (21.1)		
*Hypertensive medications*			
CCBs, *n* (%)	198 (26.3)	76 (17.4)	<0.001
Nitrates, *n* (%)	87 (11.6)	39 (9.0)	0.153
β-blockers, *n* (%)	253 (33.6)	132 (30.3)	0.212
Renin inhibitors, *n* (%)	44 (5.8)	16 (3.7)	0.093
ACE inhibitors, *n* (%)	333 (44.2)	111 (25.5)	0.001
ARBs, *n* (%)	127 (16.9)	57 (13.1)	0.077
α_1_-blockers, *n* (%)	18 (2.4)	5 (1.2)	0.096
Diuretics, *n* (%)	189 (25.1)	54 (12.4)	<0.001
Body mass, kg	86.6 ± 15.2	78.5 ± 14.0	<0.001
Body mass index, kg/m^2^	29.7 ± 4.6	27.0 ± 3.8	<0.001
Total CHOL, mg/dL	162 ± 39	186 ± 43	<0.001
HDL CHOL, mg/dL	50 ± 16	58 ± 16	<0.001
LDL CHOL, mg/dL	89 ± 35	108 ± 35	<0.001
HbA1c, %	7.4 ± 3.4	5.7 ± 2.5	<0.001
Creatinine, mg/dL	1.0 ± 0.3	0.9 ± 0.2	0.026
eGFR, mL/min/1.73 m^2^	81.9 ± 21.0	81.7 ± 17.4	0.835
Systolic BP, mmHg	136 ± 18	133 ± 17	0.001
Diastolic BP, mmHg	77 ± 10	77 ± 9	0.953
MAP, mmHg	97 ± 12	95 ± 10	0.076
Heart rate, beats/min	63 ± 11	58 ± 9	<0.001
IMT, mm	0.92 ± 0.22	0.88 ± 0.22	0.004
Lumen diameter, mm	6.5 ± 0.9	6.3 ± 0.9	<0.001
WLR, au	0.144 ± 0.039	0.142 ± 0.038	0.370

Data are shown as means ± SD, median (interquartile range) or number (%). ACE, angiotensin converting enzyme; ARBs, angiotensin receptor blockers; BP, blood pressure; CCBs, calcium channel blockers; CHOL, cholesterol; CVD, cardiovascular disease; DM+, people with type 2 diabetes; DM−, people without type 2 diabetes; DPP-4, dipeptidyl peptidase-4; eGFR, estimated glomerular filtration rate; GLP-1, glucagon-like peptide-1; HbA1c, hemoglobin A1c; HDL, high-density lipoprotein; HTRx, pharmacological treatment for hypertension; IMT, common carotid artery intima-media thickness; LDL, low-density lipoprotein; MAP, mean arterial pressure; T2DM, type 2 diabetes; WLR, common carotid artery wall-to-lumen ratio.

**Figure 1.**
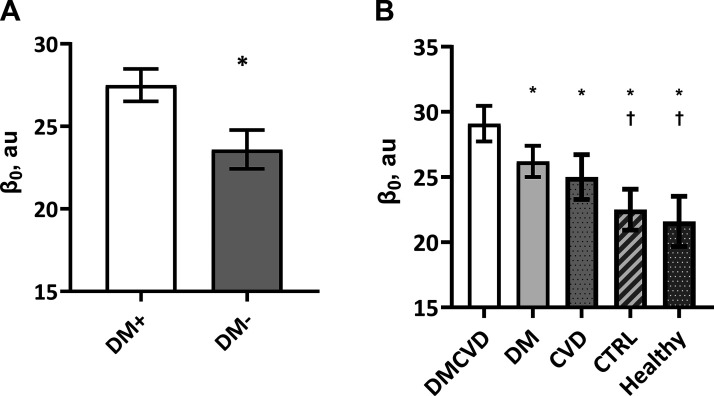
Comparisons of β_0_ between people with and without type 2 diabetes (*A*), and between people with and without type 2 diabetes stratified by a history of cardiovascular disease (*B*). *A*: data are shown as means (95% confidence intervals) after adjusting for age and sex. *Significantly different from DM+. DM+, people with type 2 diabetes; DM−, people without type 2 diabetes. *B*: data are shown as means (95% confidence intervals) after adjusting for age, sex, mean arterial pressure, and heart rate. *Significantly different from DMCVD. †Significantly different from DM. CVD, people with cardiovascular disease only (*n* = 200); CTRL, people with neither type 2 diabetes nor cardiovascular disease (*n* = 236); DM, people with type 2 diabetes only (*n* = 426); DMCVD, people with type 2 diabetes with cardiovascular disease (*n* = 327); Healthy, “healthy” people (*n* = 74).

β_0_ and CFPWV in DM+ and DM− are shown in Fig. 1 and Table 2. β_0_ was significantly greater in DM+ than in DM− after adjusting for age and sex [Fig. 1*A* 27.5 (26.6–28.3) vs. 23.6 (22.4–24.8) au, *P* < 0.001]. Further adjustment for MAP and HR did not alter the finding. Similarly, CFPWV was significantly greater in DM+ than that in DM− after adjusting for age and sex (Table 2, *P* < 0.001). Further adjustment for MAP and HR did not alter the finding. These observations remained when each cohort was stratified by sex (Table 3).

**Table 2.** Comparisons of CFPWV between people with and without type 2 diabetes

	DM+ (*n* = 753)	DM− (*n* = 436)	*P*
CFPWV, m/s	11.1 (10.9–11.3)	10.3 (10.1–10.5)	<0.001

Data are means (95% confidence intervals). CFPWV, carotid-femoral pulse wave velocity; DM+, people with type 2 diabetes; DM−, people without type 2 diabetes.

**Table 3.** Comparisons of β_0_ and CFPWV between people with and without type 2 diabetes stratified by sex

	DM+	DM−	*P*
β_0_, au			
Male	28.3 (27.2–29.4)	23.5 (22.0–25.0)	<0.001
Female	27.3 (25.8–28.8)	21.2 (19.4–23.0)	<0.001
CFPWV, m/s			
Male	11.3 (11.1–11.6)	10.3 (10.0–10.6)	<0.001
Female	11.2 (11.0–11.4)	10.6 (10.3–10.8)	<0.001

Data are means (95% confidence intervals) after adjusting for age. CFPWV, carotid-femoral pulse wave velocity; DM+, people with type 2 diabetes; DM−, people without type 2 diabetes.

Figure 1*B* and Table 4 also show β_0_ and CFPWV, respectively, between DM+ and DM− stratified by the history of cardiovascular disease (CVD) at baseline. β_0_ was greater in DM+ with CVD [29.1 (27.7–30.4) au] compared with all other groups after adjusting for age, sex, MAP and HR [DM+ without CVD, 26.2 (25.0–27.4); DM− with CVD, 25.0 (23.3–26.7) au; DM− without CVD, 22.5 (20.9–24.0) au]; β_0_ was also greater in DM+ without CVD than in DM− without CVD. Likewise, CFPWV was greater in DM+ with CVD compared with all other groups after adjusting for age, sex, MAP, and HR and was also greater in DM+ without CVD than in DM− without CVD.

**Table 4.** Comparisons of CFPWV between people with and without type 2 diabetes stratified by a history of cardiovascular disease

	DMCVD (*n* = 327)	DM (*n* = 426)	CVD (*n* = 200)	CTRL (*n* = 236)	Healthy (*n* = 74)	*P*
CFPWV, m/s	11.4 (11.1–11.6)	10.9 (10.7–11.1)*	10.6 (10.3–10.9)*	10.1 (9.8–10.4)*†	9.9 (9.6–10.3)*†‡	<0.001

Data are means (95% confidence intervals) after adjusting for age, sex, mean arterial pressure, and heart rate. In the CTRL group, the data excluding people with hypertension are presented as the “healthy” group. *Significantly different from DMCVD. †Significantly different from DM. ‡Significantly different from CVD. CFPWV, carotid-femoral pulse wave velocity; CTRL, people with neither type 2 diabetes nor cardiovascular disease; CVD, people with cardiovascular disease only; DM, people with type 2 diabetes only; DMCVD, people with type 2 diabetes with cardiovascular disease.

When people with hypertension were excluded from the DM− without CVD group to constitute an apparently “healthy” group (Healthy CTRL) in Fig. 1*B*, β_0_ was significantly lower [21.6 (19.7–23.5) au] than that in DM+ with/without CVD and showed a tendency to be lower than in DM− with CVD (*P* = 0.060). CFPWV in the Healthy CTRL group was significantly lower than in the other three groups (Table 4).

Partial correlation analyses were performed to explore associations of β_0_ and CFPWV with HbA1c, WLR, MAP, and HR after controlling for age, sex, and where appropriate, MAP and HR (Table 5). In DM+, β_0_ was significantly associated with HbA1c and HR (both *P* < 0.001). In DM−, β_0_ was significantly associated with WLR (*P* = 0.007) and HR (*P* = 0.006). In DM+, CFPWV was significantly associated with HbA1c (*P* < 0.001), MAP and HR (both *P* < 0.001). In DM−, CFPWV was significantly associated with WLR (*P* = 0.014), MAP and HR (both *P* < 0.001). Replacing HbA1c with the duration of T2DM in DM+ did not alter the associations with β_0_ or CFPWV, although the strength of associations was slightly attenuated (*r* = 0.11 *P* = 0.004 for β_0_; *r* = 0.10 *P* = 0.005 for CFPWV).

**Table 5.** Partial correlation analysis of β_0_ and CFPWV with variables that may influence arterial stiffness in people with and without type 2 diabetes

	DM+		DM−	
	β_0_	CFPWV	β_0_	CFPWV
HbA1c	*r* = 0.15, *P* < 0.001	*r* = 0.14, *P* < 0.001	*r* = 0.08, *P* = 0.115	*r* = 0.09, *P* = 0.072
WLR	*r* = 0.02, *P* = 0.592	*r* = 0.01, *P* = 0.855	*r* = 0.13, *P* = 0.007	*r* = 0.12, *P* = 0.014
MAP	*r* = 0.05, *P* = 0.169	*r* = 0.33, *P* < 0.001	*r* = 0.03, *P* = 0.537	*r* = 0.29, *P* < 0.001
Heart rate	*r* = 0.23, *P* < 0.001	*r* = 0.27, *P* < 0.001	*r* = 0.13, *P* = 0.006	*r* = 0.17, *P* < 0.001

Control variables were age, sex, MAP, and heart rate (except when the analysis included MAP/heart rate). CFPWV, carotid-femoral pulse wave velocity; DM+, people with type 2 diabetes; DM−, people without type 2 diabetes; HbA1c, hemoglobin A1c; MAP, mean arterial pressure; WLR, common carotid artery wall-to-lumen ratio.

As part of exploratory analysis, additional partial correlation analyses were performed to explore associations of β_0_ and CFPWV with the inflammatory markers (IL-6, CCL3, Pentraxin 3, and hsCRP) after controlling for age, sex, MAP, and HR (Table 6). In DM+, β_0_ showed a tendency to be associated with IL-6 (*P* = 0.055), but did not associate with other inflammatory markers. In contrast, β_0_ was significantly associated with IL-6, CCL3, and hsCRP (all *P* < 0.05) in DM−. In DM+, CFPWV was significantly associated only with IL-6 (*P* < 0.05). On the other hand, CFPWV was significantly associated with IL-6 (*P* < 0.001) and CCL3 (*P* = 0.001) in DM−, and showed a tendency to be associated with hsCRP (*P* = 0.057).

**Table 6.** Partial correlation analysis of β_0_ and CFPWV with inflammatory markers in people with and without type 2 diabetes

	DM+		DM−	
	β_0_	CFPWV	β_0_	CFPWV
IL-6	*r* = 0.07, *P* = 0.055	*r* = 0.07, *P* = 0.046	*r* = 0.20, *P* < 0.001	*r* = 0.19, *P* < 0.001
CCL3	*r* = 0.01, *P* = 0.781	*r* = 0.00, *P* = 0.950	*r* = 0.16, *P* = 0.001	*r* = 0.16, *P* = 0.001
Pentraxin 3	*r* = 0.00, *P* = 0.922	*r* = 0.01, *P* = 0.842	*r* = −0.03, *P* = 0.606	*r* = −0.05, *P* = 0.362
hsCRP	*r* = 0.01, *P* = 0.778	*r* = 0.02, *P* = 0.634	*r* = 0.12, *P* = 0.024	*r* = 0.10, *P* = 0.057

Control variables were age, sex, MAP and heart rate (same as the main analysis). CCL3, chemokine ligand 3; CFPWV, carotid-femoral pulse wave velocity; DM+, people with type 2 diabetes; DM−, people without type 2 diabetes; hsCRP, high-sensitivity C-reactive protein; IL-6, interleukin 6.

### Phase 2

Of the participants who had CFPWV measured at both baseline and 3-yr follow-up visits (*n* = 366 for DM+ and *n* = 228 for DM−), there were 56 participants in DM+ and 18 in DM− who had missing covariates needed for statistical analysis. Thus, the longitudinal data were available from 310 participants in DM+ and 210 in DM−. During the follow-up period, there were 32 (10.3%) cardiovascular events [prespecified in the SUMMIT-VIP study (17)] in DM+ and 22 (10.5%) in DM−. Age at baseline was similar between DM+ and DM− (68.6 ± 7.6 vs. 67.6 ± 8.5 yr, *P* = 0.160), as was the proportion of females [104 (33.6%) vs. 83 (39.5%), *P* = 0.164] and of current smokers [29 (9.4%) vs. 12 (5.7%), *P* = 0.131]. The proportion of participants with a history of CVD was greater in DM− than in DM+ [90 (42.9%) vs. 102 (32.9%), *P* = 0.021]. The proportion of participants who had taken antihypertensive medication [228 (73.6%) vs. 109 (51.9%), *P* < 0.001] and statin therapy [219 (70.7%) vs. 107 (51.0%), *P* < 0.001] was greater in DM+ than in DM−. In addition, the proportion of anti-hypertensive medication classes used over 3 yr was not different between cohorts except renin inhibitors (DM+, 11 at baseline and 9 at follow-up; DM−, 2 at baseline and none at follow-up, *P* = 0.063 at baseline and *P* = 0.013 at follow-up between cohorts). The duration of T2DM in DM+ was similar to that in *Phase 1* [8.0 (4.9–14.0) yr].

Table 7 shows cardiovascular risk factors in DM+ and DM− at baseline, and the absolute and percentage-change at 3-yr follow-up. At baseline, body mass and body mass index were greater in DM+ than in DM−. DM+ had higher HbA1c, and lower total and HDL cholesterol than in DM−. Resting HR was faster in DM+ than in DM−. When the absolute and percentage-change in cardiovascular risk factors were compared between DM+ and DM−, there were no significant differences, except that an absolute change in HbA1c was greater in DM+ than in DM−. There was a tendency for a greater change in serum creatinine in DM+.

**Table 7.** Baseline cardiovascular risk factors and change (absolute and percentage) at 3-yr follow-up in people with and without type 2 diabetes

	DM+ (*n* = 310)				DM− (*n* = 210)				DM+ vs. DM−		
	Baseline	Follow-up	Change	%Change	Baseline	Follow-up	Change	%Change	*p*1	*p*2	*p*3
Body mass, kg	85.5 ± 14.2	84.4 ± 15.4	−1.1 ± 7.3	0.0 ± 0.1	76.9 ± 12.9	76.3 ± 13.1	−0.5 ± 4.0	0.0 ± 0.1	<0.001	0.336	0.429
Body mass index, kg/m^2^	29.3 ± 4.4	29.1 ± 4.6	−0.3 ± 2.5	0.0 ± 0.1	26.6 ± 3.6	26.5 ± 3.8	−0.2 ± 1.5	0.0 ± 0.1	<0.001	0.462	0.635
Total CHOL, mg/dL	161.5 ± 40.2	161.6 ± 42.1	0.1 ± 31.6	0.7 ± 8.1	187.3 ± 41.4	184.7 ± 42.0	−2.6 ± 33.7	0.1 ± 7.1	<0.001	0.360	0.378
HDL CHOL, mg/dL	50.8 ± 14.3	51.5 ± 15.6	0.8 ± 8.3	0.9 ± 6.7	59.4 ± 16.8	60.9 ± 17.9	1.6 ± 8.8	1.3 ± 6.1	<0.001	0.307	0.542
LDL CHOL, mg/dL	86.7 ± 34.8	87.4 ± 36.1	1.5 ± 26.8	339.4 ± 2306.0	108.9 ± 35.5	107.2 ± 35.9	−2.2 ± 30.7	70.2 ± 1176.7	<0.001	0.151	0.128
HbA1c, %	7.3 ± 1.2	6.4 ± 2.0	1.2 ± 2.1	0.7 ± 3.5	5.7 ± 0.4	5.2 ± 1.3	1.6 ± 1.3	0.8 ± 3.3	<0.001	0.012	0.717
Creatinine, mg/dL	1.0 ± 0.3	1.0 ± 0.3	0.0 ± 0.2	0.0 ± 0.0	0.9 ± 0.2	0.9 ± 0.2	0.0 ± 0.1	0.0 ± 0.0	0.083	0.057	0.067
eGFR, mL/min/1.73m^2^	80.3 ± 18.8	78.5 ± 21.5	−1.8 ± 14.3	0.0 ± 0.2	81.7 ± 17.9	80.8 ± 19.8	-1.0 ± 10.7	0.0 ± 0.1	0.403	0.442	0.506
Systolic BP, mmHg	135 ± 18	137 ± 18	2.2 ± 17.5	2.5 ± 13.0	133 ± 17	134 ± 20	1.5 ± 17.7	1.7 ± 13.6	0.182	0.645	0.505
Diastolic BP, mmHg	75 ± 10	75 ± 9	−0.2 ± 8.6	−0.8 ± 11.8	76 ± 9	76 ± 9	−0.5 ± 8.6	−1.3 ± 11.6	0.280	0.682	0.647
MAP, mmHg	95 ± 11	96 ± 11	0.6 ± 10.6	1.3 ± 11.2	95 ± 10	95 ± 11	0.2 ± 10.4	0.7 ± 10.9	0.926	0.629	0.516
Heart rate, bpm	61 ± 11	63 ± 12	2.1 ± 9.3	4.1 ± 15.5	57 ± 8	60 ± 10	3.1 ± 8.2	6.1 ± 15.1	<0.001	0.201	0.147
IMT, mm	0.90 ± 0.22	0.93 ± 0.24	0.03 ± 0.14	4.2 ± 13.8	0.89 ± 0.22	0.91 ± 0.20	0.02 ± 0.13	3.8 ± 13.4	0.365	0.563	0.735
Lumen diameter, mm	6.3 ± 0.9	6.4 ± 0.9	0.1 ± 0.6	0.6 ± 7.5	6.3 ± 1.0	6.3 ± 1.0	0.0 ± 0.4	−0.4 ± 5.5	0.375	0.129	0.108
WLR, au	0.15 ± 0.07	0.15 ± 0.04	0.00 ± 0.07	3.9 ± 17.1	0.14 ± 0.04	0.15 ± 0.04	0.01 ± 0.03	4.4 ± 15.9	0.451	0.473	0.726

Data are shown as means ± SD. The data in the “Change” columns are presented as an absolute change, and in the “%Change” columns as a percentage change in each group following 3-yr follow-up. *p*1, *P* value after comparing baseline data between the groups; *p*2, *P* value after comparing an absolute change at follow-up between the groups; *p*3, *P* value after comparing a percentage change at follow-up between the groups; BP, blood pressure; CHOL, cholesterol; DM+, people with type 2 diabetes; DM−, people without type 2 diabetes; eGFR, estimated glomerular filtration rate; HbA1c, hemoglobin A1c; HDL, high-density lipoprotein; IMT, common carotid artery intima-media thickness; LDL, low-density lipoprotein; MAP, mean arterial pressure; WLR, common carotid artery wall-to-lumen ratio.

**Figure 2.**
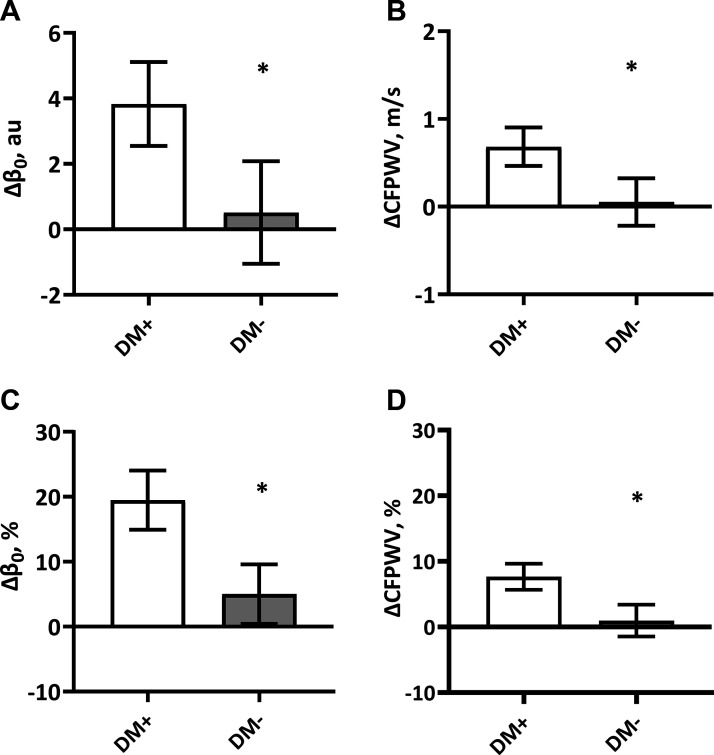
Comparisons of absolute and percentage-change in β_0_ (*A* and *C*) and in CFPWV (*B* and *D*) over 3 years in people with and without type 2 diabetes. Data are shown as means (95% confidence intervals) after adjusting for age, sex, and baseline β_0_ for the change in β_0_ and for age, sex, and baseline CFPWV for the change in CFPWV. *Significantly different from DM+. Δβ_0_, change in β_0_; ΔCFPWV, change in carotid-femoral pulse wave velocity; DM+, people with type 2 diabetes; DM−, people without type 2 diabetes.

In Fig. 2, absolute and percentage-change in β_0_ and CFPWV at 3-yr follow-up are compared between DM+ and DM−. The absolute change in β_0_ was significantly greater in DM+ than in DM− [Fig. 2*A*, 3.8 (2.6–5.1) vs. 0.5 (−1.1 to 2.1) au, *P* = 0.002] after adjusting for age, sex, and baseline β_0_. The percentage-change in β_0_ was also significantly greater in DM+ than in DM− [Fig. 2*C*, 19.5 (14.9–24.0) vs. 5.0 (−0.6 to 10.6) %, *P* < 0.001] after the same adjustments. Both absolute and percentage-change in β_0_ remained greater in DM+ than in DM− after further adjustments for change in systolic BP (or MAP) and HR. The absolute change in CFPWV was significantly greater in DM+ than in DM− (Fig. 2*B*, 0.68 (0.46–0.90) vs. 0.05 (−0.22 to 0.32) m/s, *P* < 0.001) after adjusting for age, sex, and baseline CFPWV. The percentage-change in CFPWV was also greater in DM+ than in DM− [Fig. 2*D*, 7.7 (5.7–9.7) vs. 1.0 (−1.4 to 3.4) %, *P* < 0.001] after the same adjustments. Both absolute and percentage-change in CFPWV remained greater in DM+ than in DM− after further adjustments for change in systolic BP (or MAP) and HR. These observations in change in β_0_ and CFPWV between DM+ and DM− persisted when each cohort was stratified by sex (Table 8).

**Table 8.** Comparisons of absolute/percentage change in β_0_ and CFPWV at 3-yr follow-up between people with and without type 2 diabetes stratified by sex

	DM+	DM−	*P*
β_0_: absolute change, au			
Male	3.6 (2.0–5.2)	0.7 (−1.4 to 2.7)	0.026
β_0_: percentage change, %			
Male	19.4 (13.6–25.2)	5.9 (−1.5 to 13.3)	0.005
β_0_: absolute change, au			
Female	4.3 (2.1–6.5)	0.2 (−2.3 to 2.7)	0.017
β_0_: percentage change, %			
Female	19.3 (12.0–26.7)	4.1 (−4.3 to 12.4)	0.009
CFPWV: absolute change, m/s			
Male	0.7 (0.4–0.9)	0.1 (−0.2 to 0.5)	0.016
CFPWV: percentage change, %			
Male	7.6 (5.2, 10.1)	1.8 (−1.4, 5.0)	0.005
CFPWV: absolute change, m/s			
Female	0.7 (0.3–1.1)	−0.1 (−0.5 to 0.4)	0.013
CFPWV: percentage change, %			
Female	7.8 (4.4, 11.1)	−0.2 (−4.0, 3.6)	0.003

Data are means (95% confidence intervals) after adjusting for age. CFPWV, carotid-femoral pulse wave velocity; DM+, people with type 2 diabetes; DM−, people without type 2 diabetes.

## DISCUSSION

Salient findings of this study are as follows: first, β_0_ was greater in DM+ compared with DM− at baseline. Second, β_0_ at baseline was associated with HbA1c and HR in DM+, and with WLR and HR in DM−. Finally, the increase in β_0_ over 3 yr was far greater in DM+ compared with DM−. These findings indicate that in people with T2DM, BP-independent arterial stiffness is greater and the change in arterial stiffness over time is accelerated. To our knowledge, this is the first time that accelerated changes in BP-independent arterial stiffness have been reported and indicate that in T2DM, the arterial wall is undergoing deleterious alterations to its structures and composition. Given the evidence that arterial stiffness has widespread implications for health, exacerbated change in arterial stiffness is likely to be detrimental to people with T2DM and, as such, β_0_ may provide the indication of unfavorable alterations in arterial stiffness in these patients.

### Greater BP-Independent Arterial Stiffness in T2DM at Baseline and Its Change at Follow-up

We observed greater β_0_ at baseline and a greater increase in β_0_ over 3 yr in DM+. The baseline difference provides evidence that the intrinsic stiffness of the arterial wall is altered beyond that typically seen with advancing age in the absence of T2DM. An important observation from the 3-yr follow-up is that accelerated stiffening of the large elastic arteries is an ongoing process in the presence of T2DM that represents a progressive deterioration in the functional properties of these vessels. The use of β_0_ in this study has allowed us to distinguish this change as an alteration to the BP-independent arterial stiffness that likely reflects an alteration to the intrinsic properties of the arterial wall, which, in turn, are likely to reflect alterations to the structure and composition of the artery. The use of CFPWV compliments these observations and shows that pressure-dependent arterial stiffness, a functional property of the arterial wall across physiological pressures, is altered in a similar manner. Taken together, these observations suggest that in T2DM there is an ongoing process of change to the intrinsic mechanical and likely structural properties of the arterial wall with a corresponding change in the functional stiffness of the artery under pressure.

We also note that the DM− group was a mixed group that contained patients with a history of CVD. We might expect arterial stiffness to be similar or even greater in these patients, perhaps masking differences in the DM+ group. However, the difference in arterial stiffness was sufficient to distinguish the groups. For example, the change in pressure-independent arterial stiffness over 3 yr was over 19% with the presence of T2DM but only 5% without. At baseline, we had sufficient numbers to further stratify the two main groups by history of CVD and perform an exploratory analysis of the resulting subgroups (Fig. 1*B*). The analysis indicated stepwise differences in the values for β_0_, and that β_0_ was greater in the presence of both T2DM and CVD (DM+ with CVD) than the presence of T2DM alone (DM+ without CVD). β_0_ was greater in patients with CVD (DM− with CVD) than without (DM− without CVD). The difference in β_0_ between the group with T2DM only (DM+ without CVD) and the group with CVD only (DM− with CVD) was not significant. Taken together, these data suggest that the influence of T2DM and CVD on BP-independent arterial stiffness is cumulative, with the superimposition of T2DM on CVD accelerating arterial stiffness in our cohort. The implication here is that different mechanisms are contributing to alterations to the intrinsic properties of the arterial wall in T2DM and CVD. This is consistent with previous observations that the accumulation of cardiovascular risk increases central artery stiffness (23, 24), and that there is equivalent cardiovascular risk between people with T2DM and people without T2DM but with a prior history of myocardial infarction (25, 26). We emphasize that this analysis of subgroups was undertaken post hoc and should be taken prima facie, rather than conclusive.

### Association of β_0_ with HbA1c, WLR, MAP, and HR

There was a positive association between β_0_ and HbA1c in the presence of T2DM. To our knowledge, it is unknown if glycation of hemoglobin is a useful surrogate of AGEs formation in the arterial wall of large elastic arteries, but HbA1c is a good marker of recent glycemic exposure, which is an important mechanistic component of arterial wall AGEs formation. AGEs in the arterial wall cross-link collagen molecules (9), increasing the stiffness of the material that comprises the arterial wall (i.e., alters Young’s elastic modulus in the Moens–Korteweg equation) and it seems reasonable that this structural and compositional change to the arterial wall could increase BP-independent arterial stiffness. Insulin resistance in T2DM also augments collagen synthesis, supplying “substrates” for the AGEs formation. Insulin resistance is associated with increased production of reactive oxygen species and low-grade inflammation (27, 28), and consequent reduction in NO bioavailability may also functionally increase arterial wall stiffness in T2DM. Although HbA1c is only indicative of recent glycemic exposure, the duration of diabetes was also associated with β_0_, indicating sustained exposure of the arterial wall to hyperglycemia in the DM+ group.

The absence of association between β_0_ and WLR as well as β_0_ and the inflammatory markers in DM+ might suggest a predominant contribution of AGEs to changes in the arterial wall material in this cohort. Inflammation may be an important mechanism for arterial remodeling in the absence of T2DM, but in the presence of T2DM, other contributing factors including AGEs may become more dominant and outweigh any contribution to changes in arterial wall material by inflammation. Phenotypic alterations, including vasodilatory state, and a shift of vascular smooth muscle cell from a contractile state to migratory/proliferative states in T2DM (29), might also have influenced the association between β_0_ and WLR in DM+.

β_0_ was positively associated with HR in both cohorts. Despite the well-documented influence of HR on arterial stiffness, especially on CFPWV (30, 31), the reason for this association has not been fully elucidated. Several mechanisms have been postulated, including a role for arterial wall viscoelasticity (32) and insufficient time for arterial wall recoiling (33). It is beyond the scope of this study to delineate the mechanism by which HR influences BP-independent arterial wall stiffness, but the association observed here suggests a role that should be elaborated. It is possible that the effects are acute, whereby HR is conferring some degree of functional alterations on the arterial wall. However, there could also be a chronic influence, whereby long-term exposure to a high HR stimulates structural and compositional adaptation of the wall to the consequent hemodynamic conditions.

β_0_ did not show an association with MAP in either cohort, as we expected. CFPWV was, as expected, positively associated with MAP in both cohorts, likely because of the BP-dependent nature of this measurement.

### Clinical Implications

A primary purpose of the SUMMIT-VIP study was to find intermediate vascular biomarkers that identify the risk of cardiovascular complications in T2DM and that might be useful in monitoring the response to therapy. With this in mind, we undertook this analysis to determine the utility of β_0_, as it has recently been shown to be valid (16). Our data demonstrate that β_0_ is useful for discriminating T2DM cross-sectionally as well as revealing its exacerbated increase over a 3-yr follow-up period. It brings to mind the importance of having a range of tools available for specific purposes, and β_0_ appears to be an example of a tool that might be appropriate for interventions where the properties of the arterial wall are being targeted (e.g., AGE-breakers).

### Limitations

First, the surface-distance measurement used for calculating CFPWV at the outset of the SUMMIT study was the 4-point subtraction method rather than 80% of the direct distance measurement between carotid and femoral sites that the professional societies in the field have subsequently suggested (34). Second, WLR was calculated from the common carotid artery rather than the aorta, and data should be interpreted accordingly. However, because the common carotid artery *1*) is included in the assessment of CFPWV and *2*) has been used in cardiovascular research as a surrogate artery for the aorta [for example, see Tanaka et al. (14) and Gates et al. 15)], we think its use is reasonable. Third, we acknowledge that β_0_ calculated based on CFPWV data provides the average arterial stiffness along a given path-length which includes arteries with different wall structures (35). Fourth, although the two major determinants of large artery stiffness (especially of CFPWV) are age and BP (36) that were appropriately used for statistical adjustment in this study, T2DM-related cardiovascular risk factors such as body mass index could possibly influence large elastic artery stiffness additionally to T2DM. However, the inclusion of body mass index as a covariate did not alter the difference in β_0_ and CFPWV between DM+ and DM− in this study. Fifth, the clinical utility of BP-independent arterial stiffness indices including β_0_ to predict cardiovascular events has yet to be demonstrated (37, 38). A future study should determine whether β_0_ predicts future cardiovascular events in comparison with CFPWV. Sixth, the data on menopausal status as well as hormone replacement therapy were incomplete, and thus were unable to include in this manuscript, which might have helped interpret our findings. Lastly, our observations are obtained from older adults with T2DM and therefore may not be generalizable to other populations.

### Conclusions

We demonstrate for the first time that BP-independent arterial stiffness, β_0_, is greater in people with T2DM than those without. Furthermore, β_0_ changes over 3 yr in the absence of T2DM, but it changed much more in people with T2DM. This indicates that the intrinsic properties of the arterial wall may change in a different and more detrimental way in people with T2DM and likely represent accumulation of cardiovascular risk. Because β_0_ provided complimentary data to CFPWV, we conclude that it may be a useful tool to investigate the properties of arterial wall in T2DM and for interventions that target protection or amelioration of the arterial wall.

## DATA AVAILABILITY

Data will be made available upon reasonable request.

## GRANTS

This study was supported by the European Union’s Seventh Framework Program (FP7/2007-2013) for the Innovative Medicine Initiative under grant agreement number IMI/115006 (the SUMMIT consortium) and in part by the National Institute of Health Research (NIHR) Exeter Clinical Research Facility. IG was supported by Swedish Research Council, Swedish Heart and Lung Foundation, Skåne University Hospital Foundations and the Lund University Diabetes Center–Industrial Research Center from the Swedish Foundation of Strategic Research Dnr IRC15-0067 and the Strategic Research Area Exodiab, Dnr 2009-1039). 

## DISCLAIMERS

The views expressed are those of the authors and not necessarily those of the UK National Health Service, the NIHR or the UK Department of Health and Social Care.

## DISCLOSURES

No conflicts of interest, financial or otherwise, are declared by the authors.

## AUTHOR CONTRIBUTIONS

K.A., P.E.G., and A.C.S. conceived and designed research; K.A., P.E.G., D.M.M., F.C., K.M.G., and S.V.H. performed experiments; K.A. and P.E.G. analyzed data; K.A. and P.E.G. interpreted results of experiments; K.A. prepared figures; K.A. and P.E.G. drafted manuscript; K.A., P.E.G., D.M.M., F.C., K.M.G., S.V.H., I.G., J.N., F.K., H.M.C., A.N., C.P., and A.C.S. edited and revised manuscript; K.A., P.E.G., D.M.M., F.C., K.M.G., S.V.H., I.G., J.N., F.K., H.M.C., A.N., C.P., and A.C.S. approved final version of manuscript.
